# Increase in Poison Center Reports Linked to Kratom-Containing Kava Products — National Poison Data System, United States, 2000–2025

**DOI:** 10.15585/mmwr.mm7512a1

**Published:** 2026-04-02

**Authors:** Eleanor Blair Towers, Ivy L. Williams, Christopher P. Holstege, Rita Farah

**Affiliations:** ^1^Department of Emergency Medicine, Division of Medical Toxicology, University of Virginia, Charlottesville, Virginia; ^2^Medical Scientist Training Program, University of Virginia, Charlottesville, Virginia.

**Summary**

**What is already known about this topic?**

Kava, a plant native to the Pacific Islands, is traditionally consumed in religious and cultural ceremonies. In the United States, it is sold as unregulated concentrated extracts and ready-to-drink beverages and commonly marketed as a healthy alternative to alcohol. Reports of liver toxicity and co-use with kratom, a psychoactive plant with opioid-like properties, have raised safety concerns.

**What is added by this report?**

Analysis of 2000–2025 National Poison Data System exposure data identified a resurgence of kava-related exposure reports since 2011, with recent exposures primarily involving men aged ≥20 years. Rates of serious medical outcomes have approximately doubled, coinciding with rising co-use of kava with kratom.

**What are the implications for public health practice?**

Enhanced surveillance, increased clinical awareness, and targeted education might help reduce risks from kava and its emerging co-use with kratom, particularly among U.S. men.

## Abstract

Kava (*Piper methysticum*), a central nervous system depressant derived from a plant in the pepper family native to the Pacific Islands, is traditionally consumed in religious, cultural, political, and social ceremonies. In the United States, kava emerged in the late 1990s and has experienced renewed growth and product diversification since the 2010s, with increasing availability of concentrated extracts and ready-to-drink beverages. These commercial products are commonly marketed as healthy alternatives to alcohol, sold near college campuses, and increasingly being combined with kratom, a psychoactive botanical with opioid-like effects, raising safety concerns. Data on kava-related use during January 2000–December 2025 that resulted in a report to the National Poison Data System (i.e., kava exposure report) were analyzed to assess trends by users’ demographic characteristics, exposure type, and outcomes. Kava-related exposure reports declined sharply after a 2002 Food and Drug Administration advisory on kava-associated severe liver injury but have risen steadily since 2011, reaching 203 reported exposures in 2025. Reports primarily involved adults aged ≥20 years, but demographic characteristics have changed over time. During 2000–2001, reports primarily involved females and included more children aged ≤12 years, whereas exposure reports since 2013 have predominantly involved men; reports involving children have been rare. Since 2017, reports involving combined use of kava and kratom have increased, reaching 30% (61) of all kava reports in 2025. These increases have coincided with higher rates of serious reported clinical outcomes in recent years (32% in 2025 compared with 12% in 2000). These data indicate a resurgence of overall kava exposure reports to poison centers, as well as an increase in kratom-related kava reports, which has coincided with higher rates of serious clinical outcomes. The findings in this report suggest the need for enhanced surveillance for, clinical awareness of, and public education regarding commercial products containing kava.

## Introduction

Kava (*Piper methysticum*) is a psychoactive plant native to the Pacific Islands, where its root traditionally has been prepared as a water-based beverage consumed in cultural and social settings, with relatively low reported health risk (*1*). Since the 1990s, commercial kava products have become increasingly available in the United States, including concentrated extracts, capsules, and recreational beverages sold through retail outlets and dedicated kava bars, which are often promoted as alcohol-free social venues (*2*,*3*). These commercial products are unregulated and differ substantially from traditional aqueous preparations in terms of plant material, extraction methods, and alkaloid concentration (*1*,*4*,*5*). For example, kavalactones, the primary active constituents of kava that exert sedative and anxiolytic effects, can be present in commercial products at 2–10 times the concentrations present in traditional preparations (*1*). In addition, although some clinical trials support short-term anxiolytic efficacy of traditional preparations, commercial products are often consumed without medical oversight and have been associated with gastrointestinal and neurologic adverse effects, hepatotoxicity, and drug interactions via cytochrome P450 inhibition and additive central nervous system depressant effects (*1*,*4*,*6*).

In 2002, after reports of acute liver failure and liver transplantation temporally associated with kava use, the Food and Drug Administration (FDA) warned that kava-containing dietary supplements might be associated with severe liver injury (*4*). In 2020, FDA further concluded that indiscriminate kava use is not safe for use as a recreational or relaxation beverage for human consumption (*5*). Despite these cautions, the commercial kava market has continued to expand and diversify, including the emergence of combination products including kava and kratom, a psychoactive plant with opioid-like effects (*7*). The growing availability of commercial kava products with limited available safety data underscores the need to characterize national patterns of kava use reported to U.S. poison centers to identify groups at high risk for serious clinical outcomes and guide public health practice. This report analyzes characteristics of kava-related exposures, defined as actual or suspected substance ingestion, as reported to the National Poison Data System (NPDS), for exposures during January 2000–December 2025, stratified by age, sex, and outcome.

## Methods

### Data Source and Study Design

NPDS is the national database that aggregates deidentified exposure data from all 53 U.S. poison centers. Each exposure is defined as a reported actual or suspected substance ingestion for which a poison center was consulted, regardless of toxicity or clinical manifestation. Data are submitted in near real-time after consultative services are provided to members of the general public and health care providers.

For this retrospective review, NPDS was queried for all human exposures to kava during January 1, 2000–December 31, 2025. Each exposure could involve kava alone (single-substance exposure) or in combination with other substances (multiple-substance exposure). Patient demographic data (age and sex), exposure characteristics (substances and reason for use), level of care received, clinical effect, and medical outcome were included in the analysis. The analysis was conducted on deidentified and publicly available data, and the University of Virginia Institutional Review Board determined that the research did not require human subjects review.

### Analysis

Data were descriptively analyzed. Numbers and percentages of all kava exposure reports were calculated to assess temporal trends, and kava exposure reports were stratified by sex, age, clinical effects, medical outcome, level of care received, and reported co-used substances. Age was categorized into four groups: 0–12, 13–19, 20–39, and ≥40 years. Medical outcomes were classified as serious or nonserious.^†^ Level of care was categorized as hospitalized (critical care, noncritical care, or psychiatric unit admission) or not hospitalized (treated and released, refused referral, lost to follow-up, or managed outside a health care facility). Rates for kava exposure reports were calculated per 100,000 drug exposures.

## Results

### Number and Rates of Exposures

**FIGURE 1:**
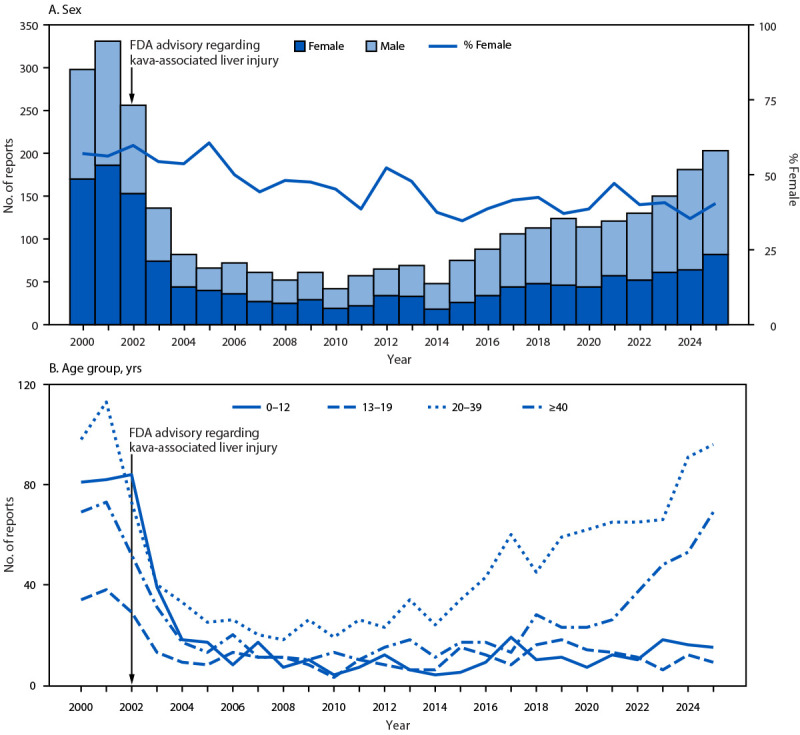
Number of kava-related reports to poison centers, by sex (A) and age group (B) — National Poison Data System, United States, 2000–2025* **Abbreviation:** FDA = Food and Drug Administration. * In 2002, FDA released a consumer advisory indicating that kava-containing dietary supplements might be associated with severe liver injury.

During the entire study period (2000–2025), a total of 3,101 kava-related exposures were reported. Before the 2002 FDA advisory, 298 and 331 annual case exposures were reported during 2000 and 2001, respectively, corresponding to approximately 34 kava-related exposure reports per 100,000 drug exposure reports (Figure 1). Coinciding with the advisory, annual exposure reports began to decline, reaching a low of 42 in 2010 (87% decrease during 2001–2010), before beginning to rise in 2011, increasing 383% by 2025 (from 57 to 203). Exposure report rates in 2010 declined to three per 100,000 drug exposure reports, but began to rise in 2011, increasing 220%, from five to 16 per 100,000 drug exposure reports in 2025.

### Demographic Characteristics of Persons with Kava Exposures

In 2000–2001, females accounted for the majority of exposure reports (56%–57%), and a substantial proportion (25%–27%) involved children aged ≤12 years (Figure 1). During 2002–2025, the percentages of all exposures involving females and children aged ≤12 years declined, reaching 40% and 7%, respectively, in 2025. During both periods (2000–2001 and 2002–2025), adults aged ≥20 years accounted for the largest percentage of exposure reports (annual average = 66%; range = 41%–81%).

### Outcomes

**FIGURE 2:**
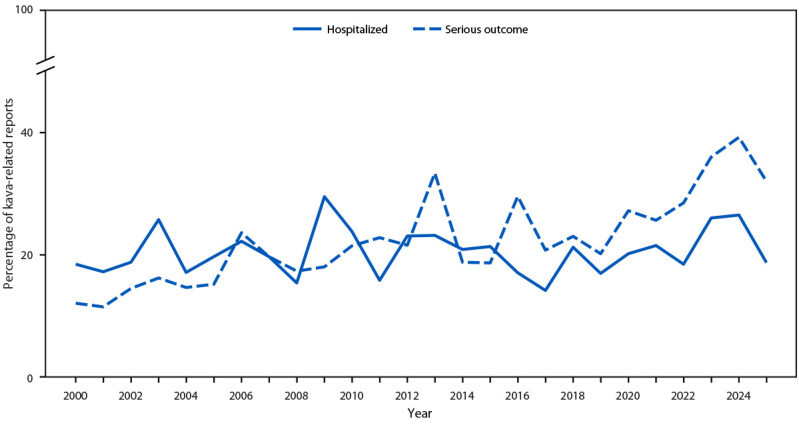
Percentage of kava-related poison center reports involving hospitalization or other serious medical outcomes* — National Poison Data System, United States, 2000–2025 * Death, major effect (life-threatening effect or one that results in substantial disability or disfigurement [e.g., status epilepticus]), or moderate effect (pronounced, prolonged, or systemic effect that usually requires some form of treatment, but is not life-threatening [e.g., hypoglycemia with confusion]).

Across the analytic period (2000–2025), an average of 20% of exposed persons were hospitalized each year (range = 14%–30%), with no apparent temporal trends (Figure 2). In contrast, the percentage of exposures associated with serious medical outcomes increased from 12% in 2000 to a high of 39% in 2024. Eight deaths were reported during the analytic period, including one each in 2000, 2001, 2005, 2017, 2023, and 2024, and two in 2021 (fatality rate = 0.25%).

### Substance Co-Exposure

**FIGURE 3:**
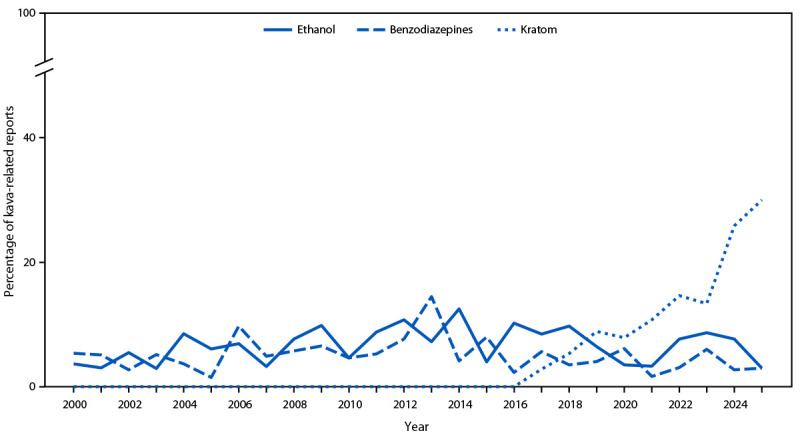
Percentage of kava-related poison center reports involving ethanol, benzodiazepines, or kratom use — National Poison Data System, United States, 2000–2025

During 2000–2025, a total of 1,347 (43%) kava-related exposure reports involved multiple substances. The most common co-involved substances were ethanol (annual average = 7%; range = 3%–13%) and benzodiazepines (annual average = 5%; range = 2%–14%) (Figure 3). However, in 2017, kratom emerged as a common co-exposure and, by 2019, kratom surpassed ethanol and benzodiazepines in multiple-substance kava-related exposures. This coincided with increasing availability of products containing kava and kratom (Supplementary Figure 1). In 2025, co-use of ethanol or benzodiazepines accounted for 3% of multiple-substance exposures, and kratom accounted for 30%.

### Reported Clinical Effects

The most common clinical effects among single-substance exposures (1,754) were gastrointestinal (vomiting and nausea), neurologic (drowsiness or lethargy, dizziness or lethargy, and agitation), and cardiovascular (tachycardia) signs and symptoms (Supplementary Figure 2). Multiple-substance exposures involving only kava and kratom (128) had similar symptomatology; however, neurologic effects included seizures and tremor, and cardiovascular effects included hypertension. Liver injury was less common for both exposure types. Moderate elevations in aspartate transaminase or alanine transaminase (>100 to ≤1,000 IU/L) were reported in 29 of 1,754 (1.7%) single-substance exposures and eight of 128 (6.3%) multiple-substance exposures involving kava and kratom.

## Discussion

Poison center surveillance functions as a national early warning system for emerging substance-related harm. After a sharp decline in kava-related exposures reported to NPDS after the 2002 FDA advisory on kava-associated liver injury (*4*), reported exposures have increased substantially since 2011. This increase parallels the expanded availability of commercial kava products in the United States (*2*,*7*), despite the FDA conclusion that these recreational or relaxation beverages are not safe for human consumption (*5*). Recent exposures have predominantly involved men aged ≥20 years and have been characterized by higher rates of serious medical outcomes, associated with increasing reports of co-use with kratom.

The resurgence of kava-related exposures reported to poison centers also coincides with the expansion of the nonalcoholic beverage market, as consumers increasingly seek alternatives to alcohol (*8*). These products are frequently sold online and in vape shops near college campuses (*9*), mirroring demographic patterns observed in exposure data, and are commonly marketed as a way to feel social without alcohol or a hangover. NPDS data findings are consistent with FDA reports indicating that kava ingestion can cause substantial gastrointestinal and neurologic effects, particularly when used in nontraditional preparations that contain higher doses or are combined with other substances such as kratom (*4*,*5*). In contrast to some countries where kava products are subject to regulatory dose limits (e.g., a maximum daily intake of 250 mg kavalactones from water-based extracts and 125 mg limit per individual tablet or capsule), kava and kava-kratom combination products sold in the United States are unregulated and advertised to contain >250 mg kavalactones per serving (30 ml), often with multiple servings per container (*5*). Actual kavalactone content might be even higher, because these products are not subject to standardized manufacturing or content verification as is alcohol. Furthermore, hepatotoxicity and other adverse effects have been linked to the chronic consumption of high-potency commercial products (*4**,**5*). In this analysis, transaminase elevations were reported in a small proportion of cases; however, no cases of acute liver failure were identified. These findings are consistent with rare reports of idiosyncratic hepatotoxicity associated with kava use. Continued promotion of these products without adequate verification of product content, consumer education regarding potential adverse health effects, and clinical awareness of evolving exposure patterns represents an ongoing public health concern.

### Limitations

The findings in this report are subject to at least four limitations. First, NPDS is subject to reporting bias, with the accuracy of data being subject to correct input and coding of medical outcomes and the highest level of care received. Second, because reporting to poison centers is voluntary, NPDS likely does not include all kava-related exposures, particularly those that are mild or self-managed. Third, poison centers upload deidentified data to NPDS; therefore, it is not possible to distinguish between first-time and repeat exposure reports. Finally, NPDS generic codes do not permit distinction among product types. Therefore, it was not possible to capture information on product type, including preparation method or dose, limiting conclusions about formulation- and dose-specific risk.

### Implications for Public Health Practice

The reemergence of kava-related exposures with clinical effects reported to poison centers in the United States, coinciding with increased availability of commercial products with high kavalactone content and co-use with kratom, represents a public health concern. Continued surveillance and increased clinical awareness could improve recognition, reporting, and characterization of high-risk product formulations and use patterns. Targeted public health education might help inform consumers that commercially available kava products are not regulated and are not recognized as safe for use as recreational beverages by FDA, and that the formulations commonly sold in the United States have been associated with adverse health effects, especially when used in large quantities. These efforts are particularly important for young men, who represent a disproportionate share of recent exposures.

## Footnotes

† Serious outcomes: death, major effect (life-threatening effect or one that results in substantial disability or disfigurement [e.g., status epilepticus]), or moderate effect (pronounced, prolonged, or systemic effect that usually requires some form of treatment but is not life-threatening [e.g., hypoglycemia with confusion]). Nonserious outcomes: minor effect (minimal effect that resolves rapidly [e.g., sinus tachycardia without hypotension]), no effect, unrelated effect, or not followed (no follow-up based on clinical judgment that the exposure was likely nontoxic or expected to result in only minimal, trivial clinical effects).

## References

[1] Abbott P. Kava: a review of the safety of traditional and recreational beverage consumption. Rome, Italy: Food and Agriculture Organization of the United Nations; World Health Organization; 2016. https://openknowledge.fao.org/server/api/core/bitstreams/f97fbe5f-244e-4301-be2a-03ac39ef630b/content

[2] Bian T, Corral P, Wang Y, Kava as a clinical nutrient: promises and challenges. Nutrients 2020;12:3044. 10.3390/nu12103044 33027883 PMC7600512

[3] Wakim O. Atlanta kava bars offer island culture, alcohol-free social spaces. Atlanta Journal-Constitution [Internet]. Atlanta, GA: Atlanta Journal-Constitution; 2025. https://www.ajc.com/food-and-dining/2025/12/atlanta-kava-bars-offer-island-culture-alcohol-free-social-spaces/

[4] Food and Drug Administration. Consumer advisory: kava-containing dietary supplements may be associated with severe liver injury. Silver Spring, MD: US Department of Health and Human Services, Food and Drug Administration; 2002. https://wayback.archive-it.org/7993/20170722144010/https:/www.fda.gov/Food/RecallsOutbreaksEmergencies/SafetyAlertsAdvisories/ucm085482.htm.

[5] Food and Drug Administration. Scientific memorandum: kava (review of the published literature pertaining to the safety of kava for use in conventional foods). Silver Spring, MD: US Department of Health and Human Services, Food and Drug Administration; 2020. https://www.fda.gov/media/169556/download

[6] Sarris J, LaPorte E, Schweitzer I. Kava: a comprehensive review of efficacy, safety, and psychopharmacology. Aust N Z J Psychiatry 2011;45:27–35. 10.3109/00048674.2010.522554 21073405

[7] Pont-Fernandez S, Kheyfets M, Rogers JM, Smith KE, Epstein DH. Kava (*Piper methysticum*) in the United States: the quiet rise of a substance with often subtle effects. Am J Drug Alcohol Abuse 2023;49:85–96. 10.1080/00952990.2022.2140292 36410029

[8] Purcell D. Innovation and artisan options driving opportunity in adult nonalcoholic beverages. Good Company Launch Pad. Washington, DC: US Chamber of Commerce; 2024. https://www.uschamber.com/co/good-company/launch-pad/nonalcoholic-beverage-startups

[9] Sun DL, Schleicher NC, Recinos A, Henriksen L. Spatial clustering of hookah lounges, vape shops, and all tobacco retailers near colleges. Nicotine Tob Res 2022;24:834–9. 10.1093/ntr/ntac007 35022769 PMC9048933

